# First detection of mutated *ERG11* gene in vulvovaginal *Candida albicans* isolates at Ouagadougou/Burkina Faso

**DOI:** 10.1186/s12879-022-07619-5

**Published:** 2022-08-08

**Authors:** Essi Etonam Dovo, Théodora Mahoukèdè Zohoncon, Sessi Frida Tovo, Serge Théophile Soubeiga, Isabelle Touwendpoulimdé Kiendrebeogo, Albert Théophane Yonli, Rogomenona Alice Ouedraogo, Amana Mètuor Dabire, Florencia Wendkuuni Djigma, Christelle Wendyam Nadembega, Marius Belemgnegre, Paul Ouedraogo, Dorcas Obiri-Yeboah, Jacques Simpore

**Affiliations:** 1 Molecular Biology and Genetics Laboratory (LABIOGENE), University of Joseph Ki ZERBO, BP 7021, Ouagadougou 03, Burkina Faso; 2 Research Institute for Health Sciences (IRSS), 03 BP 7047, Ouagadougou 03, Burkina Faso; 3 Biomolecular Research Center Pietro Annigoni (CERBA), 01 BP 364, Ouagadougou 01, Burkina Faso; 4 University of Dedougou, BP 176, Dedougou, Burkina Faso; 5 Saint Camille Hospital of Ouagadougou (HOSCO), 01 BP 444, Ouagadougou 01, Burkina Faso; 6 Department of Microbiology and Immunology, School of Medical Sciences, University of Cape Coast, PMB, Cape Coast, Ghana

**Keywords:** *ERG11* gene, *Candida albicans*, Azole resistance, Vulvovaginitis, Burkina Faso

## Abstract

**Background:**

Vulvovaginal candidiasis is an important cause of morbidity among women due to *Candida* species. In the last decades, resistance to azoles, first-line antifungals has increased. One molecular mechanism of azole resistance by *Candida* involves mutations in the *ERG11* gene encoding lanosterol 14-α-demethylase, the target enzyme. This study was conducted to identify the clinical *Candida* species associated in vulvovaginal candidiasis; to determine the rate of antifungal resistance among *Candida albicans* isolates and to determine mutated *ERG11* gene at Saint Camille Hospital in Ouagadougou, Burkina Faso.

**Methods:**

Antifungals susceptibility were performed using Kirby–Bauer disk diffusion method. *ERG11* gene was detected using conventional PCR in *C. albicans* isolates resistant to at least one azole.

**Results:**

Out of 262 clinical strains isolated, *C. albicans* accounted for 59.90%, followed by *Candida glabrata* 27.86%, *Candida famata* 7.25%, *Candida tropicalis* 3.05% and *Saccharomyces cerevisiae* 1.91%. Resistance rate of fluconazole to *C. albicans* was 59.54%. *ERG11* gene was found in 9.79% of 92 *C. albicans* strains resistant to azoles.

**Conclusions:**

This detection of mutated *ERG11* gene in *C. albicans* is the first in Burkina Faso and may be a cause of azole resistance in recurrent *Candida* vulvovaginitis.

**Supplementary Information:**

The online version contains supplementary material available at 10.1186/s12879-022-07619-5.

## Background

Vulvovaginal candidiasis (VVC) is a widespread infection of genital tract caused by *Candida* species (GTIs). It is the second most prevalent vaginal infection in women of childbearing age, preceded by bacterial vaginosis. Its importance is based on the fact that it affects a woman’s social life because of varied and disturbing symptoms and high incidence [1, 2]. About 75% of adult women have at least once in life which 40–50 experience further episodes. Up to 9% of women experience at least three episodes per year, which is defined as recurrent vulvovaginal candidiasis (RVVC). The number of episodes tends to be more in women who are young, sexually active, pregnant, immunocompromised or on contraceptive pills [3]. However, *C. albicans* is the most causative agent in VVC, emerging non-*albicans Candida* (*NAC*) are too increasingly isolated [4]. The first line antifun*g*als used for treatment are azoles. Azoles inhibit the activity of lanosterol 14-α-demethylase (*Erg11p*) encoded by *ERG11*gene. *Erg11p* is a target enzyme which regulates a rate-limiting step in the ergosterol biosynthetic pathway. Ergosterol is an essential sterol component of fungal cell membranes. In the last decades, antifungals specially azole resistance has increased. In Burkina Faso, recent studies have shown a prevalence of more than 50% of azole resistance [5–8]. Several molecular mechanisms are involved in azoles resistance of *C. albicans*: (i) alterations in the affinity of lanosterol 14α-demethylase to azoles due to mutations or overexpression of the *ERG11* gene (the commonest mechanism). It cause a structural change in lanosterol 14a-demethylase sequence. As a consequence, the affinity between azoles and the target enzyme may be decreased, leading to the resistance to azole; (ii) Reduction of intracellular azole due to the overexpression of efflux drugs. Efflux drugs are mediated by membrane transport proteins belonging to the family of transporters of the ATP binding cassette ABC, (*CDR1* and *CDR2*) or to the main facilitator superfamily (*MDR1* and *FLU1*); (iii) Changes in the cell wall or plasma membrane; and also (iv) Formation of *Candida* biofilm which provide a protecting econiche [9].

In Burkina Faso, there is a lack of data available about antifungals resistance and its molecular mechanisms in *C. albicans*. Hence, this study aims to investigate *Candida* species distribution and their prevalence to antifungal resistance; and to determine the mutated *ERG11* gene in resistant *C. albicans* isolated in vulvovaginitis at Ouagadougou, Burkina Faso.

## Methods

### Study setting

**Fig. 1:**
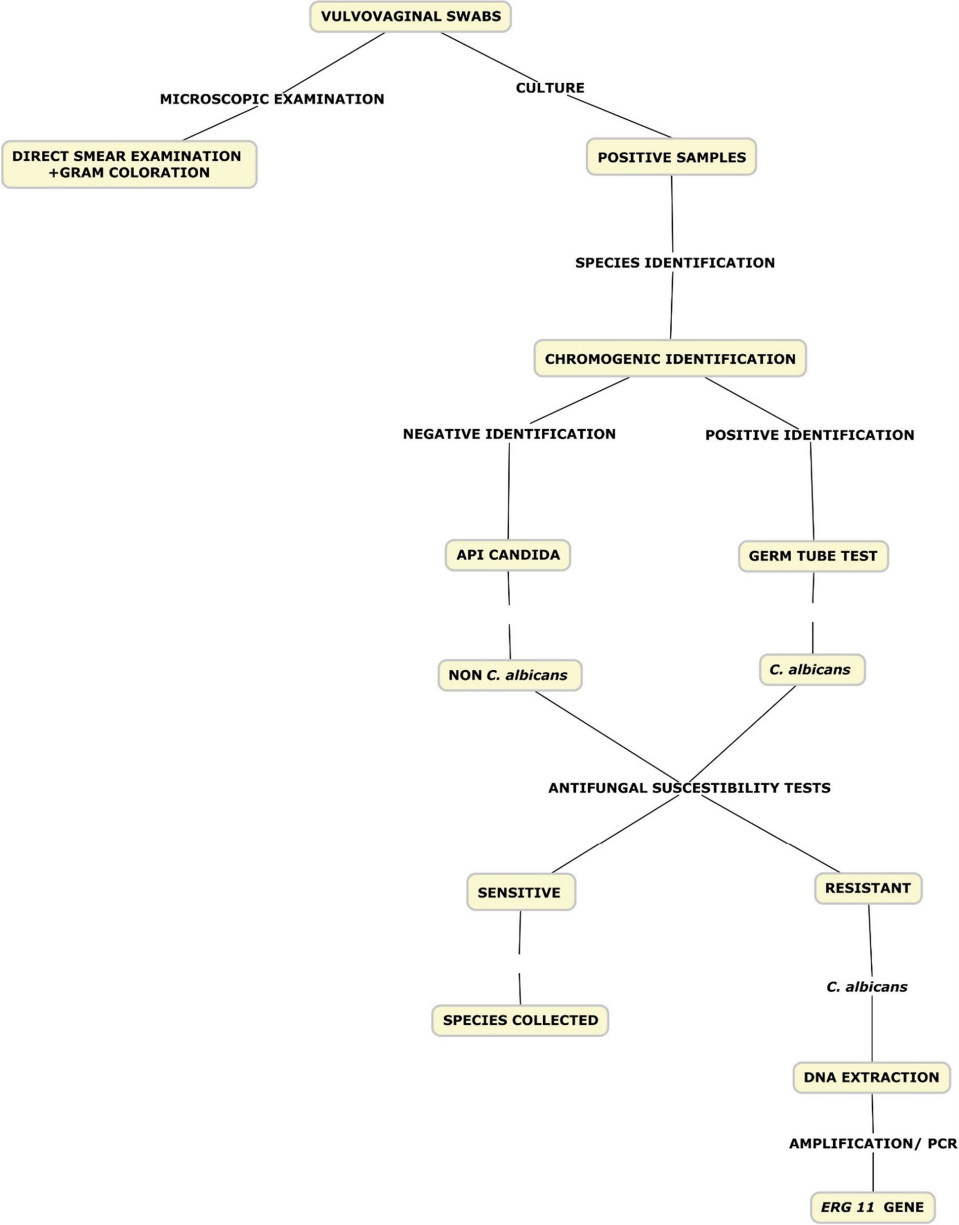
Flow chart of all procedures used

This is a cross-sectional study that took place from October 2018 to March 2020. The *Candida* strains isolated were obtained from women received for routine vulvovaginal swabs examination at Bacteriology Laboratory of Saint Camille Hospital of Ouagadougou (HOSCO), Burkina Faso. The detection of the *ERG11* gene was carried out at the Pietro Annigonni Biomolecular Research Center (CERBA/LABIOGENE) in Ouagadougou. The study was approved by the Institutional Ethics Committee of HOSCO/CERBA for the collection of clinical samples, materials and methods. All the procedures used in the present study are shown in a flow chart (Fig. 1).

### Sample Collection

Two high vaginal swabs were collected for each woman. For sexually women, a sterile vaginal speculum was placed into the vagina. The vaginal swabs were obtained by inserting sterile cotton wool swab into the posterior vaginal fornix and rotated gently. For virgins, the speculum was not placed. One of the swabs was used for direct smear examination and the second swab for cultures.

### Identification of *Candida* species

All vaginal swabs were streaked onto Sabouraud Dextrose Agar (SDA) supplemented with chloramphenicol (REF 610103 Liofilchem R© srl Italy) and incubated at 37 °C/24–48 h. Once cultures positives, the strains were purified and identified using chromatophilic medium ChromID® *Candida* Agar (REF 43 639, BioMérieux, Marcy l’Etoile, France), API® *Candida* (REF 10 500, BioMérieux, Marcy l’Etoile, France), Apiweb Standalone version 1.3.2. and the Germ tube test [10, 11].

### Antifungal susceptibility testing

Antifungal sensitivity test was assessed by the Kirby–Bauer disk diffusion method according to the recommendations of CLSI M44-A/EUCAST for yeasts [12]. The inoculum suspension was prepared in 5 mL of NaCl saline solution and the turbidity adjusted to 0.5 McFarland standard. Antifungals (Liofilchem R© srl, Italy): Clotrimazole CLO (50 µg), Econazole ECN (10 µg), Ketoconazole KCA (10 µg), Miconazole MCL (10 µg), Fluconazole FLU (100 µg), Itraconazole ITR (50 µg) and Nystatin NY, had been used and the incubation at 37 °C for 24–48 h. Inhibition zones had been measured in millimeters and the results interpreted using interpretive breakpoints according to the recommendations of CLSI/EUCAST validated for in vitro sensitivity tests (Table 1) [12, 13].

**Table 1:** Antifungals interpretive breakpoint

Antifungus	Interpretive breakpoint	Diameters (mm)
Nystatine	S	> 10
	R	< 10
Fluconazole, Itraconazole	S	≥ 19
	SDD	15–18
	R	14
Econazole, Clotrimazole, Miconazole, Ketoconazole	S	≥ 20
	SDD	10–20
	R	≤ 10

*S* sensitive; *SDD* susceptible dose dependent; *R* resistant

### DNA extraction

The fungal DNA was extracted basing on boiling–freezing method as described by Da Silva et al. [14]. In an Eppendorf tube, 8–10 fresh colonies of *C. albicans* were mixed in 0.5 mL of Luria Bertani LB broth. To release the genetic material, the LB broths were vortexed, heated at 100 °C in a water bath for 15 min and cooled to room temperature. DNA was then precipitated in 250 µL of absolute ethanol and washed three times in 1 ml of 75% cold ethanol. After that, it was dried on a hot plate and resuspended in 100 µL of sterile water.

### PCR amplification

The *ERG11* gene was detected by conventional PCR with a very specific primer which covers the entire open reading frame. This primer was designed to detect any changes in the *ERG11* gene apparently associated with resistance due to exposure of *C. albicans* to azoles. The primer sequence was F: 5′-CAA GAA GAT CAT AAC TCA AT-3″, R: 5′-AGA ACA CTG AAT CGA AAG-3″ [15]. Amplification was performed on the GeneAmp System PCR 9700 Thermocycler (Applied Biosystems, CA, USA) using Amplitaq Gold master mix: Buffer, 10×; DNA polymerase 5 U/µL; 125 mM MgCl_2_ and 10 mM of a mix of dNTP. The PCR was carried out in a reaction volume containing master mix 12.5 µL; primer F 1 µL; primer R 1 µL; DNA 4 µL and H_2_O 6.5 µL. An internal positive control consisting of the DNA of a *C. albicans* strain resistant to all the azoles and having the mutant *ERG11* gene and a negative control were included in each series. The amplification program was used: Initial denaturation at 95 °C for 10 min; followed by 35 cycles of denaturation at 95 °C for 30s, Hybridization at 53 °C for 30s and elongation at 72 °C for 2 min; then a final elongation 72 °C for 7 min.

### Revelation of PCR products

Electrophoresis on 0.8% agarose gel (prepared in 1× tris base-borate EDTA solution) at 70 V for 1 h 35 min was used to separate the amplicons. A 1 kb DNA marker was used as a molecular weight index. The 1640 bp PCR products were visualized with ethidium bromide (BET) (0.5 µg/mL) under UV using the developer (GENE FLASH).

## Results

### Study population

The study population was component of 498 women aged 11–54 years with mean age 28.6 ± 6.71. Positive cultures were obtained from 256/319 (80.33%) women with abnormal vaginal samples. The women were grouped into three according to their ages as follows: < 25 years; 25–35 years; > 35 years.

### Species identification

**Fig. 2:**
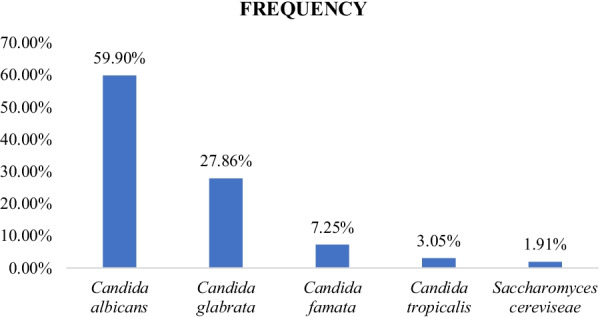
Frequency of *Candida* species isolates

In our study, 262 clinical strains of *Candida* isolates were collected from patients (aged 11–47 years) suspected of having vulvovaginal *candidiasis* (VVC). They consisted of 157 *Candida albicans* (*C. albicans*) (59.9%), 73 *Candida glabrata* (*C. glabrata*) (27.86%); 19 *Candida famata* (*C. famata*) (7.25%), 8 *Candida tropicalis* (*C. tropicalis*) (3.05%) and also 5 *Saccharomyces cerevisiae* (*S. cerevisiae*) (1.91%) (Fig. 2). The distribution of *Candida* species according to the age of the patients is shown in Table 2.

**Table 2:** Distribution of species isolated according to age groups

Species	Age of patients (years) N (%)				
	X ≤ 24	25–35	X > 35	Total	p
	N = 148 (29.7)	N = 238 (47.8)	N = 112 (22.5)	N = 498 (100)	
*C. albicans*	55 (11.0)	75 (15.5)	27 (5.4)	157 (31.5)	< 0.0001
*C. glabrata*	32 (6.4)	28 (5.6)	13 (2.6)	73 (14.7%)	0.013
*C famata*	5 (1.0)	13 (2.6)	1 (0.2)	19 (3.8)	0.002
*C. tropicalis*	2 (0.4)	5 (1)	1 (0.2%)	8 (1.6)	0.20
*S. cerevisiae*	2 (1.4)	0 (0)	3 (0.1)	5 (1.0%)	0.001

### Antifungal susceptibility tests

The results of Antifungal susceptibility testing for the 262 clinical isolates of *Candida* are shown in Table 3.

**Table 3:** Summary table of antifungal susceptibility tests

	Azoles						Polyens
	CLO n (%)	KCA n (%)	MCL n (%)	ECN n (%)	FLU n (%)	ITR n (%)	NY n (%)
S	148 (56.5)	168 (64.1)	151 (57.6)	229 (87.4)	106 (40.5)	171 (65.3)	247 (94.3)
SDD	17 (6.5)	33 (12.6)	22 (8.4)	12 (4.6)	50 (19.1)	56 (21.4)	–
R	97 (37)	61 (23.3)	89 (34.0)	21 (8)	106 (40.4)	35 (13.3)	15 (5.7)

*S* sensitive; *SDD* susceptible dose dependent; *R* resistant; *n* number of *Candida* isolates; *CLO* Clotrimazole; *KCA* Ketoconazole; *MCL* Miconazole; *ECN* econazole; *FLU* Fluconazole; *ITR* Itraconazole; *NY* Nystatin

According to the Clinical and Laboratory Standards Institute definitions, the antifungal susceptibility test showed that all families tested in this study were affected by resistance. In fact, among azoles tested, 156 isolates (59.54%) had reduced susceptibility to Fluconazole (106 R and 50 SDD) followed by 114 isolates (43.51%) to Clotrimazole (97 R and 17 SDD). 229 *Candida* isolates were active to Econazole (87.4%) followed by 171 isolates to Itraconazole 65.26%. Among the polyenes tested, 247 isolates (94.27%) were very sensitive to Nystatin (Table 3).

### Antifungal susceptibility tests of different species

*C. albicans* isolates were resistant to Clotrimazole (36.3%) and Fluconazole (35.0%). *C. glabrata* isolates were resistant to Fluconazole (49.3%) and Clotrimazole (39.7%). Fluconazole (52.6%) and Miconazole (42.1%) had the strongest resistance against *C. famata* isolates. All *C. famata* isolates were susceptible to Econazole. Econazole and Clotrimazole were the most active against *C. tropicalis* isolates at 87% and 62.5% respectively. All species were very sensitive to Econazole. Among polyenes, Nystatin was very active on all the strains isolates, more particularly on the strains of *C. famata* and *C. tropicalis* where no resistance to Nystatin had been observed. The correlation between azoles antifungal sensitivity and *Candida* species were shown in Table 4.

**Table 4:** Azoles antifungal sensitivity tests of different species

	CLO N (%)	KCA N (%)	MCL N (%)	ECN N(%)	FLU N(%)	ITR N(%)	Total	p
*C. albicans*	95 (36.3)	103 (39.3)	92 (35.1)	138 (52.7)	79 (30.2)	115 (43.9)	157 (31.5)	< 0.0001
*C. glabrata*	38 (14.5)	49 (18.17)	41 (15.6)	62 (23.7)	17 (6.5)	41 (15.6)	73 (14.7%)	< 0.0001
*C. famata*	8 (3.1)	8 (3.1)	10 (3.8)	18 (6.9)	7 (2.7)	9 (3.4)	19 (3.8)	0.13
*C. tropicalis*	5 (1.9)	4 (1.5)	5 (1.9)	7 (2.7)	2 (0.8)	4 (1.5)	8 (1.6)	0.69
*S. cerevisiae*	2 (0.8)	4 (1.5)	3 (1.1)	4 (1.5)	1 (0.4)	2 (0.8)	5 ( 1.0%)	0.73

*S* sensitive; *N* number of species isolates; *CLO* Clotrimazole; *KCA* Ketoconazole; *MCL* Miconazole; *ECN* Econazole; *FLU* Fluconazole; *ITR* Itraconazole

### Antifungal susceptibility tests in *C. albicans*

**Fig. 3:**
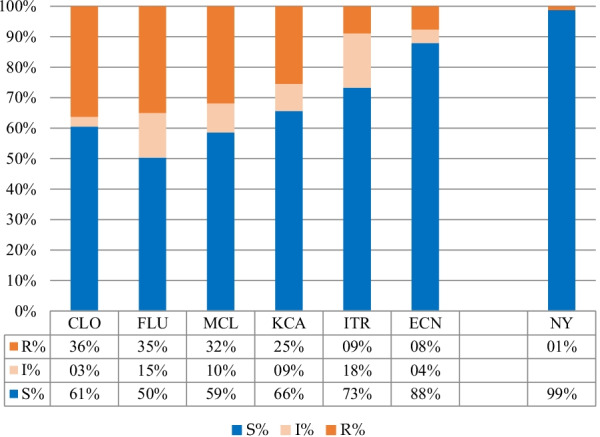
Frequency of antifungal susceptibility tests in *C. albicans*. *S* sensitive; *SDD* susceptible dose dependent; *R* resistant; *CLO* Clotrimazole; *KCA* Ketoconazole; *MCL* Miconazole; *ECN* Econazole; *FLU* Fluconazole; *ITR* Itraconazole; *NY* Nystatin

In total, *92 C. albicans* isolates (58.59%) were resistant to azoles. The results showed that in *C. albicans*, Clotrimazole, Fluconazole, and Miconazole had the highest resistance respectively at 36.30%; 35%; 31.81%. Figure 3 summarize the results of the sensitivity tests of *C. albicans* to azoles and polyen.

Out of 92 *C. albicans* isolates, 18 (19.56%) were resistant to azoles had co-resistance to Clotrimazole–Fluconazole–Miconazole. On the other hand, 23 isolates (25.05%) had co-resistance to Clotrimazole–Fluconazole.

### Determination of mutated *ERG11* gene

**Fig. 4:**
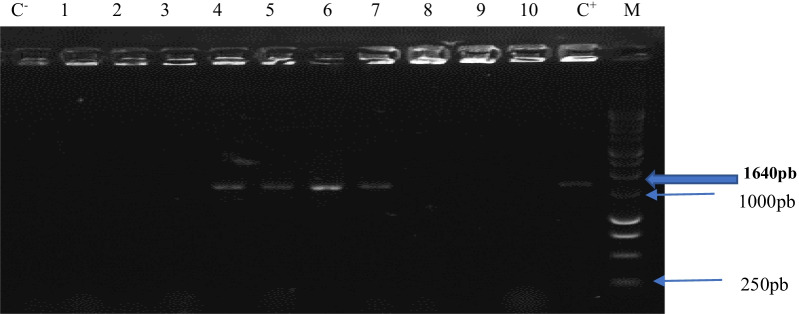
Agarose gel electrophoresis of the amplicon lane. *M* DNA marker; *C*^+^ positive control; *C*^−^ negative control and lanes 4, 5, 6 and 7 showing visible amplification of *ERG11* gene with band size of 1640 bp for resistant isolates of *C. albicans*

Only strains of *C. albicans* having at least one resistance to azoles were selected for the detection of the *ERG11* resistance gene. In total, the DNAs of 92/157 (58.59%) of the *C. albicans* strains were tested. Nine (09) strains (9.79%) of *C. albicans* exhibited the *ERG11* resistance gene. Figure 4 show detection of bands obtained during visualization under UV after electrophoresis on agarose gel.

Among the strains presenting the mutated *ERG11* gene, 55.55% were resistant to Clotrimazole, 44.45% had resistance to Fluconazole; 44.44% had co-resistance to Fluconazole + Miconazole; 33.34% were co-resistant to Fluconazole + Clotrimazole + Miconazole. Note that all the strains with the presence of the *ERG11* gene had co-resistance.

## Discussion

Several studies on vulvovaginal candidiasis have shown that *C. albicans* is the commonest species isolated. In our study, out of 262 *Candida* clinical isolates, *C. albicans* remains to be the most common species at 59.90% followed by *Candida glabrata* 27.86%. This results are similited to Zida et al. study who obtained a prevalence of 59.36% of *C. albicans* [8] and also Kouadio-Yapo et al., a high prevalence of 64.8% in Ivory Coast [16].

In our study, younger women, between 11 and 24 years had a lower prevalence 35.1% of vulvovaginal candidiasis, while the prevalence was 47.8% in the 25–35 age group. In every age group, *C. albicans* has the highest prevalence: 57.29% in 11–24 years; 62.0 in 25–35 years and 60.01% in over 35 years which are statistically signifiant. It is similar to Chuku et *al* study with a prevalence rates of 52.03% recorded among women who were within the age group of 21–30 years [15]. It is in concordance with the findings of Nadembega et al. who reported a prevalence rates of 59% of *C. albicans* in a study of vaginal infections in women aged 15–24 in Ouagadougou [7]. This may be due to many risks factors such as hormonal influences, predominant nutritional types or sexual activities. The high prevalence of C. *albicans* isolates may be due to its ability of adaptation to the human being which constitutes its main reservoir and its virulence factors such as colonization of human tissues, biofilm formation, hyphae formation as reported by Gonçalves et al. [1].

Sangaré et al., in their study on *Candida* species isolated from pregnant women in Burkina Faso, a prevalence rate of 40.39% for *C. albicans* and a rate of 59.61% for NCA with *C. glabrata* (32.69%) followed by *Candida tropicalis* (*C. tropicalis*) (15.38%) and *Candida krusei* (*C. krusei*) (11.54%) [6]. We did not isolate *C. krusei* in our study. In China, a study conducted by Xiang et al. showed that *C. albicans* isolates were 50% followed by 18% *C. glabrata*, 17% *C. parapsilosis*, 11% *C. tropicalis* and 2% *C. krusei* [17]. Similar studies have shown that the prevalence rates of other species are variable from one study population to another, from one region to another but *C. glabrata* is the most isolated on the non-*C. albicans* species [18–21]. This may be attributed to the ability of *C. glabrata* to adapt and survive in macrophages as an immune evasion strategy. The incidence of non-*C. albicans* species such as *C. glabrata*, *C. tropicalis*, *C. parapsilosis*… has increased probably due to the use of narrow-spectrum antifungals only act on *C. albicans* [19, 22].

We isolated *S. cerevisiae* at 1.91%. This is extremely rare. In South America, Papaemmanouil et al. study obtained a rate prevalence of 2.17% on recurrent vaginal candidiasis in sexually active adult women [23]; in Asia, Guo et al. obtained similar prevalence rate of 2.3% in their study on *Candida* involved in vaginitis and their susceptibility tests to antifungals [24]. This could also be one of the causal agents of recurrent CVV.

In our study, results of the antifongigram are similarly to those of Zida et al., in 2017 where Fluconazole was resistant at 66.5% and Nystatin very active on *C. albicans* at 94.7% (p < 0.05). Kouadio-Yapo et al. in 2017, obtained resistance rate of 39.7% for Itraconazole and a rate of 26.3% for Fluconazole (p < 0.05) at Pasteur Institute of Ivory Coast. This difference can be explained by the fact that Fluconazole is the widely used molecule among azoles. In recent years, many studies have shown resistance of *C. albicans* and NCA to azoles specifically to Fluconazole [25]. In the United States, a study on the resistance of *Candida* to azoles in vaginal infections have shown 11% in *C. glabrata* (p < 0.05) [26]. In our study, we obtained co-resistance in *C. albicans* isolates, a prevalence rate of 25% of co-resistance to Clotrimazole–Miconazole and 19.56% of co-resistance to Clotrimazole–Fluconazole–Miconazole. Our results differ from those of Das et al. and Farhan et al., who obtained respectively co-resistance for Fluconazole–Ketoconazole and for Clotrimazole–Ketoconazole [27, 28]. In our study, 25.5% of strains of *C. albicans* were resistant to Ketoconazole. All this testify the global distribution of resistance to azoles and the very great variability of their prevalence according to country, biological samples and species; and therefore, a global public health problem.

In our study, the *ERG11* gene was detected in *C. albicans* isolates resistant to azoles. In Nigeria, similar studies were carried out showing the presence of the *ERG11* gene at 11.18% in strains of *Candida* resistant to Fluconazole isolated from vulvovaginitis (p > 0.05) and 88.89% in *C. albicans* strains all resistant to the azoles Fluconazole and Voriconazole isolated only in pregnant women [29]. In China, studies on *ERG11* gene mutations in *C. albicans* isolated in vulvovaginitis (p < 0.05) showed a prevalence of the detected gene of 8.4% [17] and 12.2% [30]; which is similar to ours. In United States, White et al. obtained a prevalence of 10.52% of the *ERG11* gene by sequencing in their study on the molecular mechanisms of resistance of *C. albicans* to azoles [31]. Our results can be explained by the fact that there are other mechanisms of azoles resistance which are not yet explore in our country.

Because of the toxicity of others antifungal drugs and the multiples *Candida* Drugs Resistant, there is a need for new antifungal agents for the efficient management of *C. albicans* infections [32]. Natural drugs, microbial natural products from plants have shown their efficiency on *Candida* strains. Their antifungal mechanisms are: interaction with ergosterol, inhibition of the synthesis of cell wall components, inhibition of sphingolipid synthesis and inhibition of protein synthesis. Natural products from plants mostly exert their antifungal effects by membrane-active mechanism [33].

Our study was limited to find relationship between *Candida* burden with/without *ERG11* mutation and comorbidities/factors because of the lack of patients informations. Due to the non-availability of sequencing in our environment, the specify mutations in *ERG11* gene were not determine (Additional file 1).

## Conclusions

As demonstrated in this study, *C. albicans* not only cause vulvovaginal candidiasis but also non-*C. albicans* species such as *C. glabrata* which are pathogenic too. The high prevalence rate of azole resistance indicate the necessity of culturing any *Candida* species isolated and doing their antifungal sensitivity tests to manage treatments guidelines for more efficiency. Therefore, vulvovaginal candidiasis are not to be considered anymore as a trivial disease. The surveillance of antifungal resistance patterns and investigation of other mechanisms of azole resistance in all *Candida* isolates is recommended.

## Supplementary Information

**Additional file 1: Figure S1.** Agarose gel electrophoresis of the amplicon lane. **Table S1.** Azoles antifungal susceptibility tests of different species. **Table S2.** Azoles antifungal susceptibility tests of different species.

## Data Availability

The datasets used and/or analyzed during the current study are available from the corresponding author on reasonable request.

## Abbreviations

*ERG*: *Ergosterol gene*
CERBA: Biomolecular Research Center Pietro Annigoni
LABIOGENE: Molecular Biology and Molecular Genetics Laboratory
HOSCO: Saint Camille Hospital of Ouagadougou

## Declarations

### Statistical analyses

Standard descriptive analysis was performed using Excel 2016 and IBM SPSS 25.0 (SPSS Inc., Illinois, USA) software. The χ^2^ test and Fisher’s exact test was used to compare categorical variables and the differences between groups were considered statistically significant for p ≤ 0.05.
