# Supplementary material for: First detection of mutated ERG11 gene in vulvovaginal Candida albicans isolates at Ouagadougou/Burkina Faso
Source: BMC Infect Dis. 2022 Aug 8;22:678. doi: 10.1186/s12879-022-07619-5 (PMC9361531; doi:10.1186/s12879-022-07619-5)
Supplement: Supplementary file 1 — Additional file 1: Figure S1. Agarose gel electrophoresis of the amplicon lane. Table S1. Azoles antifungal susceptibility tests of different species. Table S2. Azoles antifungal susceptibility tests of different species. [file 12879_2022_7619_MOESM1_ESM.docx]

C^-^ 22 21 20 19 18 17 16 15 14 13 12 11 10 9 8 7 6 5 4 3 2 1 C^+^ M


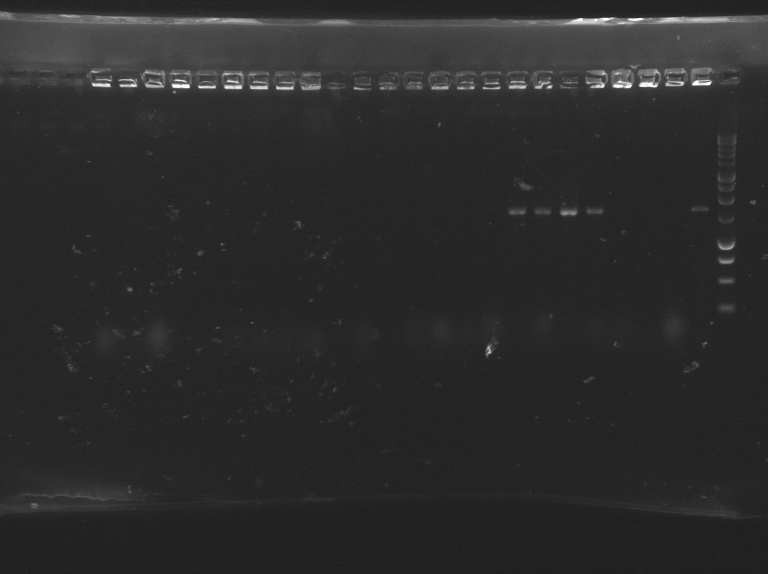


250pb

**1640pb**

1000pb

**Figure S1:** Original Agarose gel electrophoresis of the amplicon lane

Legend : M: DNA marker; C^+^ positive control; C^-^ negative control and lanes 4,5,6 and 7 showing visible amplification of *ERG11* gene with band size of 1640 bp for resistant isolates of *Candida albicans*

**Table S1 : Azoles antifungal susceptibility tests of different species**

|  | **CLO**  **n (%)** | | | **KCA**  **n (%)** | | | **MCL**  **n (%)** | | | **ECN**  **n (%)** | | | | **FLU**  **n (%)** | | | | **ITR**  **n (%)** | | |
| --- | --- | --- | --- | --- | --- | --- | --- | --- | --- | --- | --- | --- | --- | --- | --- | --- | --- | --- | --- | --- |
|  | S | SDD | R | S | SDD | R | S | SDD | R | S | SDD | R | S | | SDD | R | S | | SDD | R |
| ***C. albicans*** | 95 (60,5) | 5 (3,2) | 57 (36,3) | 103 (65,6) | 14 (8,9) | 40 (25,5) | 92 (58,6) | 15 (9,6) | 50 (31,8) | 138 (87,9) | 7 (58,3) | 12 (7,6) | 79 (50,3) | | 23 (14,6) | 55 (35,0) | 115 (73,2) | | 28 (17,8) | 14 (8,9) |
| ***C. glabrata*** | 38 (52,1) | 6 (8,2) | 29 (39,7) | 49 (67,1) | 11 (15,1) | 13 (17,8) | 41 (56,2) | 5 (6,8) | 27 (37,0) | 62 (84,9) | 4 (33,3) | 7 (9,6) | 17 (23,3) | | 20 (27,4) | 36 (49,3) | 41 (56,2) | | 23 (31,5) | 9 (12,3) |
| ***C. famata*** | 8 (42,1) | 6 (31,6) | 5 (26,3) | 8 (42,1) | 6 (31,6) | 5 (26,3) | 10 (52,6) | 1 (5,3) | 8 (42,1) | 18 (94,7) | 1 (5,3) | 0 - | 7 (36,8) | | 2 (10,5) | 10 (52,6) | 9 (47,4) | | 3 (15,8) | 7 (36,8) |
| ***C. tropicalis*** | 5 (62,5) | 0 - | 3 (37,5) | 4 (50,0) | 1 (12,5) | 3 (37,5) | 5 (62,5) | 1 (12,5) | 2 (25,0) | 7 (87,5) | 0 - | 1 (12,5) | 2 (25,0) | | 2 (25,0) | 4 (50,0) | 4 (50,0) | | 1 (12,5) | 3 (37,5) |
| ***S. cerevisiae*** | 2 (40,0) | 0 - | 3 (60,0) | 4 (80,0) | 1 (20) | 0 - | 3 (60,0) | 0 - | 2 (40,0) | 4 (80,0) | 0 - | 1 (20,0) | 1 (20,0) | | 3 (60,0) | 1 (20,0) | 2 (40,0) | | 1 (20,0) | 2 (40,0) |

**Legend:** S: Sensitive; SDD: Susceptible Dose Dependent; R: Resistant; n: Number of *Candida* isolates; CLO: Clotrimazole; KCA: Ketoconazole; MCL: Miconazole; ECN: Econazole; FLU: Fluconazole; ITR: Itraconazole;

**Table S2 :** **Polyens antifungal susceptibility tests of** **different species**

|  | ***C. albicans*** | | ***C. glabrata*** | | ***C. famata*** | | ***C. tropicalis*** | | ***S. cerevisiae*** | |
| --- | --- | --- | --- | --- | --- | --- | --- | --- | --- | --- |
| **NY n (%)** | ***S*** | ***R*** | ***S*** | ***R*** | ***S*** | ***R*** | ***S*** | ***R*** | ***S*** | ***R*** |
|  | 155 (98,7) | 2 (1,3) | 69 (94,5) | 4 (5,5) | 19 (100,0) | 0 | 8 (100,0) | 0 | 4 (80,0) | 1 (20,0) |

**Legend:** S: Sensitive; SDD: Susceptible Dose Dependent; R: Resistant; n: Number of *Candida* isolates; NY: Nystatin
